# Analysis of pre-service teachers’ argumentation-based academic writing process

**DOI:** 10.3389/fpsyg.2022.1040332

**Published:** 2022-12-05

**Authors:** Bekir Direkci, Serdar Akbulut, Bilal Şimşek, Mevlüt Gülmez, Emel Nalçacıgil Çopur

**Affiliations:** Turkish Education Department, Faculty of Education, Akdeniz University, Antalya, Turkey

**Keywords:** language education, writing education, academic writing, argumentation, mixed method research design

## Abstract

The purpose of this research is to examine the participating students’ argumentation-based academic writing processes and the contributions of these processes to the students’ academic writing skills. The participants of the study, which was conducted through mixed method research design, were a group of 53 pre-service Turkish Teachers who are in their first year of Turkish education program at a state university in Turkey during the 2020–2021 academic year. In this research, the data were obtained through student products, rubrics, reflective participant diaries, and a semi-structured interview form. SPSS 23 was used in the analysis of the quantitative data, and NVivo 12 programs were used in the analysis of qualitative data. When the results of the analyses are considered in general, it can be deduced that academic writing practices based on argumentation contributed to the development of students in the dimensions of “subject and content,” “organization,” “language use,” “citation,” and “writing process.” In the data obtained from the reflective diaries and interviews, although some difficulties were pointed out, the statements of the students regarding their development came to the fore. In addition, it was pointed out that argumentation contributes not only to students’ academic writing skills, but also their development of thinking, objectivity, research motivation, and critical thinking.

## Introduction

Academic articles are texts in which the findings obtained from scientific research carried out for a specific purpose are explained in an academic style (Demirci, 2014). These texts are created for many purposes, such as reporting research, solving a problem, testing a hypothesis, discussing a topic, and giving information about it, and synthesizing the studies done by different researchers (Bailey, 2011; Dinçer, 2018). Academic writing, on the other hand, is the documentation of the research process and its results (Monippally and Pawar, 2010). According to Bahar (2014), academic writing is defined as the reporting process of a scientific research. The reporting process aforementioned is among the most frequently encountered problems in the conduct of scientific research (Kozak, 2018).

Within the scope of reporting, problems related to developing a thesis statement, making connections between sentences, writing coherent paragraphs, organizing ideas, and the text, elaborating the argument, having problems with word choice and sentence structure, citing sources and reorganizing citations have been pointed out in existing publications (Pablo and Lasaten, 2018; Ivanova, 2020; Lin and Morrison, 2021; Aldabbus and Almansouri, 2022). Within this framework, teaching academic writing includes purposes such as creating the text based on scientific foundations and various evidences; being able to deal with the results obtained as a result of one’s own work together with the results of other studies; gaining the ability to conduct research around established standards; being able to see from different perspectives; being able to present a text that is consistent in terms of arguments and content; and being linguistically proficient.

Academic writing should not be seen as just a writing skill. For a qualified study, first of all, an adequate literature review should be done, evidence should be sought for the relevant study and the data obtained should be written down. Thus, scientific studies provide progress in the light of the results of previous research (Kozak, 2018). For this reason, the data to be obtained within the framework of the research and data obtained from different studies should be assimilated and both sets of data should be synthesized. In this process, the concept of “citation” comes into prominence. Citation is one of the distinguishing indicators for academic papers (Coffin, 2009) in which information or finding is associated with a particular source (Swales, 2014; Kafes, 2017; Zhang, 2022). Citation, one of the main features of academic articles, is among effective strategies authors use to support their proposals (Hyland, 1999, p. 362). With this strategy, the author provides evidence for his argument and enriches his/her research with different perspectives. During the citation stage, there are points that the researcher should pay attention to formally and semantically. The author should use the citations in his/her own research by synthesizing it. Clear linking of ideas is important to help readers follow the text (Swales and Feak, 2012). This can be achieved with correct synthesis and the use of correct language. Consequently, in academic writing, the writer must support ideas well, order them logically, justify them rigorously, and weave these ideas together tightly (Fang, 2021). Academic language, on the other hand, is an objective and qualified language that has different functions from everyday speech, requires high-level thinking skills, and is guided by knowledge and technical terms (Sarıkaya, 2020). A scientific style is used in the academic writing process, and certain conclusions are reached by asserting reasons and evidence instead of definite judgments (Gillett et al., 2009). In academic writing, the author’s opinion should be clear, s/he should support the arguments s/he put forward with the evidence, prove the accuracy of the statements s/he put forward in the text, and should prefer a convincing and causal style (Caine, 2008). In this respect, the argumentative narrative style has an important place in academic writing. In the argumentative narrative, the author’s opinion is revealed to be valid and valuable by presenting the reasons related to the subject, credible reasons and sufficient evidence (Beyreli and Konuk, 2018). The reasons put forward by the author are the elements that show how his claim can be successfully defended against attacks and how the counterclaim can be refuted (Van Eemeren et al., 2004). In addition, justifications are one of the basic elements that contribute to the development of the argument. The development of the argument presented in an academic text is considered an important feature of successful writing (Lea and Street, 1998). Furthermore, many students have been seen to be either unaware of developing an argument in their writing or have difficulty in doing so (Davies, 2008; Bacha, 2010). In this context, it is very important to develop students’ argumentation skills and to use this in academic writing processes. It is necessary to use high-level reasoning and thinking skills in order to create a comprehensive scientific knowledge about a subject (Topdemir and Unat, 2014, p. 7). At the stage of creating scientific knowledge, researchers use scientific arguments to explain the experiment and observation processes (Bakırcı et al., 2017). In this process, researchers frequently use argumentation, which requires asking questions, making claims, and supporting their claims with evidence (Erduran and Jimenez-Aleixandre, 2007; Shi, 2019). The framework of argumentation, which is a structured scientific argumentation technique, was introduced by Toulmin (1958) and was handled by different researchers as a technique for justifying a claim with evidence and solving an existing problem (Jiménez-Aleixandre et al., 2000; Cho and Jonassen, 2002). The overlap between the nature of scientific research and the “data-claim-justification” process, which is at the core of argumentation, highlights the relationship between these two concepts (Uluçınar Sağır and Kılıç, 2013).

When the studies in the literature are reviewed, it is seen that argumentation has positive impacts on the areas such as academic achievement (Canoz et al., 2022), concept teaching (Boyraz et al., 2016), higher-level thinking skills (Erkek and Bostan, 2019; Viyanti et al., 2020), critical thinking and decision-making skills (Tonus, 2012; Giri and Paily, 2020), problem-solving skills (Kardaş, 2013), argumentation creation (Torun and Şahin, 2016), and teaching of socio-scientific issues (Deniz, 2014). However, it was determined that students had difficulties in producing arguments (Jonassen and Kim, 2010) and using elements such as data-justification-support and rebuttal to strengthen their claims (Jiménez-Aleixandre et al., 2000; Kuhn, 2010). As a matter of fact, the inability to verify the asserted claims with data is seen as one of the obstacles to argumentation (Zohar and ve Nemet, 2002). Teaching these factors, which are the basic elements of creating scientific texts, and enabling students to gain argumentation skills should be an important educational goal (Crowell and Kuhn, 2012; Bağ and Çalık, 2017). However, some studies in the literature emphasized that students’ argumentation skills are low, and that sufficient attention is not paid to the development of these skills in schools (Weinstock et al., 2006; Crowell and Kuhn, 2012). In this context, it is of great importance that students gain argumentation skills, which are closely related to scientific method and academic writing.

Although academic writing is seen as an area of interest for graduate students and academics, it is very important to gain this skill at previous levels. As a matter of fact, undergraduate students are expected to conduct research on different courses and subjects, and report these research studies (Siyez, 2016). In order to meet this expectation, it is seen that the content of the Turkish Language II course, which is included in the teacher training programs developed by YÖK (Council of Higher Education), is prepared for teaching academic writing. In addition, it is important to gain these skills not only for meeting the expectation of using academic writing skills during this course, but also for different purposes in other courses from different departments. As a matter of fact, the program aims to train Turkish teacher candidates competently in scientific texts as well as in other text types. Especially, the responsibility given to Turkish teachers in the teaching of informative texts in the Milli Eğitim Bakanlığı (2019) requires Turkish teachers to be competent in this field. In this context, it is planned to teach academic writing to Turkish teacher candidates in a way that will contribute to the content of the Turkish Language II course. Within the framework of the lesson plan, pre-service teachers are expected to present their argument as a writer and develop their writing around this argument. The content of the article should be created around the argument discussed and intellectual consistency should be ensured throughout the article. In addition, the author must organize the patterns of ideas about the argument s/he put forward, give them in a logical order, and correctly construct the sentence and paragraph connections during the writing phase. The author should know the correct citation, should not make citation mistakes, and should make the citation in correct form or style. In some studies, it was found that students had difficulty in supporting their claims with evidence (Shi, 2019) and could not evaluate the evidence by associating it with their claims (Sadler, 2004; Watson et al., 2004; Acar et al., 2010) and the evidence they presented contradicted their claims, but they continued to assert their original claims (Evagorou et al., 2012). As another element of academic writing, writers are expected to use a scientific style and objective expressions and write in a fluent and understandable language in accordance with the grammatical rules of the language. While creating a scientific text, the author examines the ideas and findings of different experts and includes them in his/her own article accordingly.

Based on this context, the aim of our research is to examine argumentation-based academic writing processes and the contributions of these processes to students’ academic writing skills. The sub-problems of our research around this aim can be listed as follows:

1. What is the effect of argumentation-based academic writing practices on students’ ability to create academic texts?
2. How are academic writing practices based on argumentation reflected in student diaries?
3. What are the students’ views on academic writing practices based on argumentation?

## Materials and methods

### Research model

Mixed methods research design was used in the study (Mccrudden et al., 2019; Kelle, 2022). In the quantitative part of the study, pre-service Turkish teachers’ argumentation-based academic writings were analyzed and scored. Reflective diaries kept by prospective Turkish teachers during the argumentation-based academic writing training process, and the interviews conducted with the participants after the completion of the training process and academic writing phase constitute the qualitative data of this study.

### Study group

In this study, purposive sampling technique was used to determine the study group, and pre-service teachers who took “Turkish Language II” course and volunteered to participate in the study were included in the study group in order to ensure the development of academic writing. The study group in the experimental process of the research consisted of 53 Turkish teacher candidates, of whom 28 were female and 25 were male, who were studying in the first grade in the Turkish language teaching undergraduate program of a state university in the spring term of the 2020/2021 academic year, and who had not received any training on academic writing before. The main rationale behind the selection of the study group from Turkish teacher candidates is that they, as a mother tongue teacher, should be competent in scientific texts as well as in different text types in terms of teaching four basic language skills and developing literacy skills. Thus, an important place is given to the teaching of informative texts in the Milli Eğitim Bakanlığı (2019) for Turkish teacher candidates in this field. In addition, the fact that Turkish Language II course, which has a course content aimed at gaining academic text writing proficiency in the undergraduate program, was for the first-year students, was also effective in determining the study group.

For the reflective diaries, which constitute one part of the qualitative dimension of the research, all participants who participated in the experimental process kept a weekly diary in the determined time period. In the semi-structured interviews, the other part of the qualitative dimension, 15 Turkish language teacher candidates, of whom seven were male and eight were female, and who participated in the experimental process and volunteered for the interview, constituted the study group.

### Data collection

In this research, “student products,” “academic writing assessment rubric,” “reflective participant diaries” and “semi-structured interview form” were used as data collection tools. The descriptions of these tools are given in the following parts.

#### Student products

Academic texts written by students. The students were made to write two academic texts one before and one after the argumentation-based academic writing training. In this framework, a total of 106 written products were obtained from 53 students.

#### Academic writing assessment rubric

Rubrics are explanatory/graded scoring schemes used to evaluate the learning process of individuals or the learning product that emerges at the end of the process (Brookhart, 2018). These charts, in which the expected/targeted things are defined at each stage, can be used both as an assessment tool and as a teaching tool. While teachers can follow the learning process according to the levels in the rubrics (Arter, 2002), students can also obtain information for the next stages (Moskal and Leydens, 2000). In this study, an analytical rubric developed by Lallmamode et al. (2016) was preferred in order to evaluate the academic texts produced by the students. The rubric in question consists of academic writing and e-portfolio sections, and in this study, academic writing section consisting of 5 sub-dimensions, “subject and content, organization, language use, citation, and writing process,” was used. The rubric used in the evaluation of academic writing has a 5-stage grading system in each sub-dimension (see Table 1).

**Table 1:** Subsections and levels of the rubric used in the research.

Academic writing assessment rubric		
**Criteria**	**Lev descriptors**	**Weight**
Subject & Content	5	•highly focused & coherent • thorough & adequate development of thesis
	4	• mostly focused & coherent • good development of thesis
	3	• focused but sometimes incoherent • limited development of thesis
	2	• often unfocused & incoherent • weak development of thesis
	1	• mostly unfocused & incoherent • inadequate development of thesis
Organization	5	• very well organized throughout; clear logical sequencing • effective use of cohesive markers
	4	• well organized throughout; logical sequencing • good use of cohesive markers
	3	• adequately organized; logical but poor sequencing • limited range of cohesive markers
	2	• inadequate organization; lacks logic and poor sequencing • many inappropriate cohesive markers
	1	•serious disorganization; unclear sequence • cohesive markers almost inappropriate
Language Use	5	•writing flows smoothly; very few language errors • highly appropriate register
	4	•writing flows rather smoothly; some language errors • adequate & appropriate register
	3	• many language errors but writing comprehensible • some inappropriate register
	2	•many language errors; writing not comprehensible at times • many inappropriate register
	1	•dominated by language errors; writing mostly incomprehensible • little knowledge of register
Citation	5	• very strong ability to cite and quote accurately • accurate application of citation style
	4	• good ability to cite and quote accurately • apply citation style with occasional errors
	3	• reasonable ability to cite and quote accurately • apply citation style with some errors
	2	• weak ability to cite and quote accurately • apply citation style with many errors
	1	• very weak ability to cite and quote accurately • inaccurate application of citation style
Writing Process	5	• diligently reviewed & proofread • excellent incorporation of others’ responses/ideas
	4	• good reviewing & proofreading • very good incorporation of others’ responses/ideas
	3	• acceptable reviewing & proofreading • good incorporation of others’ responses/ideas
	2	• weak reviewing & proofreading • poor incorporation of others’ responses/ideas
	1	• poor reviewing & proofreading • insufficient proof of incorporation of others’ responses/ideas

#### Reflective participant diaries

Reflective diaries are written documents including various data (such as analysis, figure, draft, quotation, comment, and impression) in which learners chronologically bring their feelings and thoughts together with their actions such as research, inquiry, experiment, observation, suggestion, etc. (Johnson, 2014). During the research, the students filled out a diary once a week and their opinions and thoughts about the process were determined with these diaries.

#### Semi-structured interview form

After 8 weeks of experimental process of academic writing implementations, a semi-structured interview form was used to conduct the interviews in order to identify the views of the participants on the argumentation-based academic writing training.

### Implementation process

The research was carried out within the scope of the 1st year spring term “Turkish Language II” course at the Faculty of Education, Turkish Language Teaching. The reason behind choosing this course for the implementation of this research is that the content of the “Turkish Language II” course, which is a common course under the dimension of “General Knowledge” for education faculties within the framework of “Teacher Training Undergraduate Programs” [Yüksek Öğretim Kurulu Başkanlığı (YÖK), 2018], is aimed at academic writing.

Turkish Language II course content:

*Features of academic language and writing; using definitions, concepts and terms in academic writings; objective and subjective expression; structure and types of academic texts (articles, reports and scientific abstracts, etc.); making a claim, proposition (justifying, defending, or opposing an idea); formal features of scientific reports and articles; the steps of writing a report; explanation, discussion, establishing intertextual relations, citation (citing and footnotes, bibliography); writing titles, summarizing, writing keywords; ethical principles to be considered in scientific writings; academic text writing practices”* [Yüksek Öğretim Kurulu Başkanlığı (YÖK), 2018].

An 8-week plan was constructed for the training and the planned activities for the experimental process to be implemented within the scope of the research. Accordingly, in the first phase, the participants were identified and informed about the study. In the first week, in order to determine the academic writing skills of the participants, the participants were asked to write an academic text on a subject of their own choice. They were informed that they could make use of the library, and the computer laboratory in the faculty during the process of text creation. The written products of the students were evaluated in detail according to the rubric employed in the research. The results were also shared with the students, and they were informed about the evaluation criteria before writing. In addition, the texts created by the students were checked through a plagiarism detection program, and students were delivered feedback.

Between weeks 2 and 7, 6 weeks of training activities were carried out to develop argumentation-based academic writing skills. During the time frame of 6 weeks, activities were carried out on the determined topics (see Table 2). Sample texts were shown, and practices were carried out regarding weekly topics. Furthermore, students were given feedback in line with the rubric criteria. Students were also asked to keep a reflective diary during the weeks of these activities.

**Table 2:** Implementation process work-timeline.

Work-time schedule	Weeks							
	1	2	3	4	5	6	7	8
Identification of participants	X							
Preliminary information	X							
Pre-training text creation	X							
Academic text and academic writing		X						
Argumentation		X						
The place and importance of argumentation in academic writing		X						
Finding evidence for arguments			X					
Academic databases			X					
Literature review			X					
Citation rules				X				
Writing bibliography				X				
Creating a text for the argument					X			
Ensuring consistency throughout the text					X			
Organizing the text					X			
Argumentative narration						X		
Developing thesis and antithesis						X		
Language use in scientific writings						X		
Synthesizing							X	
Proofreading studies							X	
Creating post-training text								X
Evaluation								X

In the eighth week, after the training activities, students were asked to create academic texts without any subject restriction. They were informed that they could benefit from the library, and computer laboratories in the faculty. By collecting the academic texts prepared by the students after the process, both the experimental process was completed, and post-test data were obtained. The results of the evaluation of these texts were also shared with the students and their opinions about the process were obtained. The weekly workload and the subject distribution for the implementation process of argumentation-based academic writing training are shown in Table 2.

### Analysis of data

During this research, the data obtained from the data collection tools were analyzed and interpreted in accordance with the structure of the mixed methods research design. The analysis of the data started while the implementation process of the research continued.

#### Quantitative data

At the beginning of the research process, the researchers examined the academic texts, which were the pre-test data written by the students at the beginning of the research process, and they were scored in line with the academic writing assessment rubric developed by a researcher and an academician with a PhD in Turkish education as an expert. At the end of the 8-week implementation process, the post-test academic texts written by the students were also scored by the same coders in line with the Academic Writing Assessment Rubric.

The reliability of the analysis of student products (academic texts) that were obtained during the research was demonstrated depending on the level of reliability between coders. Cohen’s Kappa formula was used to calculate reliability among coders. The data obtained in the study were coded by an expert other than the researcher, and the inter-coder agreement was calculated with kappa. According to the results (see Table 3), the fit values of the pre-test score measurements of the academic writing rubric and its sub-dimensions were 0.86 for subject and content; 71 for organization; 0.77 for language usage;78 for citation; 0.72 for writing process, and finally, 0.77 for the total. The fit values of post-test score measurements were 0.80 for subject and content; 0.82 for organization; 0.88 for language usage; 0.94 for citation; 0.80 for writing process; and for the post-test total score, the results were 0.85. These results demonstrate that the agreement between the coders was high in data coding. Any different encodings among the coders were re-evaluated by the coders and after the agreement was reached, the final scoring was calculated, and the data analysis for the experimental part of the research was carried out over the final scoring. In order to determine which statistical tests will be used in the analysis of these data, the results of the normality test of the distribution of the data were examined (see Table 4).

**Table 3:** Fict indices results.

Rubric and sub-dimensions	Kappa
Subject and content pre-test	,86
Organization pre-test	,71
Language use pretest	,77
Citation pre-test	,78
Writing process pre-test	,72
Academic writing pre-test total	,77
Subject and content post-test	,80
Organization post test	,82
Language use post test	,88
Citation final test	,94
Writing process post test	,80
Academic writing posttest total	,85

**Table 4:** Normality test results of scores from academic writing and its sub-dimensions.

Academic writing and its sub-dimensions	Kolmogorov–Smirnov		
	Statistic	df	Sig.
Subject and content pre-test	,402	53	,000
Organization pre-test	,281	53	,000
Language use pre-test	,295	53	,000
Citation pre-test	,231	53	,000
Writing process pre-test	,357	53	,000
Academic writing pre-test total	,162	53	,001
Subject and content post-test	,355	53	,000
Organization post-test	,333	53	,000
Language use post-test	,300	53	,000
Citation final test	,211	53	,000
Writing process post-test	,264	53	,000
Academic writing post-test total	,161	53	,002

Table 4 demonstrates that according to the Kolmogorov–Smirnov test, the mean values of the academic writing pretest-posttest total score and the mean values of pretest-posttest sub-dimension (namely subject and content, organization, language use, citation and writing process) scores do not meet the normality assumption (Z = 0.402; 0.281; 0.295; 0.231; 0.357; 0.162; 0.355; 0.333; 0.300; 0.211; 0.264; 0.161; *p* < 0.05). Based on these data, non-parametric tests were selected for all analyses. In this direction, the data obtained from the student products within the scope of the study were analyzed with the Wilcoxon Signed Ranks Test.

#### Qualitative data

Apart from the first and last week activities, students were asked to keep a reflective diary of the activities carried out within the framework of the research. The reason for not keeping a diary in the first and last week was that the participants were expected to see the positive and negative aspects and deficiencies in the texts they wrote in the first week in the process, while they were also expected to reflect their views in the last week in semi-structured interviews. After the 2nd, 3rd, 4th, 5th, 6th and 7th week activities, the students were asked to keep a reflective diary for the activities of that week. Reflective participant diaries were collected weekly during the research and analyzed by the researchers using the content analysis technique. After the analysis of the reflective diaries was completed, a meeting was held in order to eliminate the differences between the coders and the differences in emerged contents and themes were resolved on the basis of unanimity and a majority of votes.

After the 8-week training implementation process and receiving academic texts from the participants as post-test data, the participants’ views on the argumentation-based academic writing process were collected through semi-structured interviews. The interviews were audio recorded so as not to lose any data. The obtained data were transcribed and analyzed by two researchers with content analysis technique using NVIVO 12 program. A meeting was held to eliminate the differences between the researchers after the analysis, and the data analysis process was completed by eliminating the differences in emerged contents and themes on the basis of unanimity/majority vote.

## Results

“What is the effect of argumentation-based academic writing practices on students’ ability to create academic texts?” The results of the pre-test and post-test applications for this research question are shown in Table 5.

**Table 5:** Descriptive statistics for academic writing pre-test and post-test measures.

	N	Minimum	Maximum	Mean	Std. deviation
Subject and content pre-test	53	1	4	2,34	,706
Organization pre-test	53	1	5	2,28	,794
Language use pre-test	53	1	5	2,18	,962
Citation pre-test	53	1	5	2,21	1,17
Writing process pre-test	53	1	4	2,26	,738
Academic writing pre-test total	53	7	18	11,28	2,87
Subject and content post-test	53	2	5	4,02	,604
Organization post-test	53	2	5	3,47	,723
Language use post-test	53	2	5	3,72	,794
Citation final test	53	2	4	3,04	,784
Writing process pos-test	53	2	5	3,53	,749
Academic writing post-test total	53	13	21	17,77	1,58

Table 5 demonstrates the total scores and for the scores of each sub-dimension, which are subject and content [x¯ (pretest) = 2.34; x¯ (posttest) = 4.02], organization [x¯ (pretest) = 2.28; x¯ (posttest) = 3.47], language use [x¯ (pretest) = 2.18; x¯ (posttest) = 3.72], citation [x¯ (pretest) = 2.21; x¯ (posttest) = 3.04)], writing process sub-dimensions [x¯ (pretest) = 2.26; x¯ (posttest) = 3.53], and academic writing total [x¯ (pretest) = 11.28; x¯ (post-test) = 17.77]. When the pre-and post-test measurement averages are compared, it is seen that the post-test measurement scores are higher. Whether this difference was statistically significant or not was examined with the Wilcoxon Signed Ranks Test, and the results are presented in Table 6.

**Table 6:** Wilcoxon Signed Ranks Test results.

Subject and content post-test-pre-test	Negative Ranks	0	,00	,00	-6,217	,000
	Positive Ranks	49	25,00	1,225,00		
	Ties	4				
	Total	53				
Organization post-test-pre-test	Negative Ranks	5	17,10	85,50	−5,185	,000
	Positive Ranks	42	24,82	1,042,50		
	Ties	6				
	Total	53				
Language use post-test-pre-test	Negative Ranks	4	14,88	59,50	−5,236	,000
	Positive Ranks	41	23,79	975,50		
	Ties	8				
	Total	53				
Citation post-test-pre-test	Negative Ranks	11	14,77	162,50	−3,676	,000
	Positive Ranks	31	23,89	740,50		
	Ties	11				
	Total	53				
Writing process post-test-pre-test	Negative Ranks	3	18,50	55,50	−5,331	,000
	Positive Ranks	42	23,32	979,50		
	Ties	8				
	Total	53				
Academic writing total post-test-pre-test	Negative Ranks	3	3,50	10,50	−6,188	,000
	Positive Ranks	49	27,91	1,367,50		
	Ties	1r				
	Total	53				

According to Table 6, the scores for sub-dimensions are participants’ subject and content (Z = −6.217; *p* < 0.01), organization (Z = −5.185; *p* < 0.01), language use (Z = −5.236; *p* < 0.01) 0.01), citation (Z = −3.676; *p* < 0.01), and writing process (Z = −5.331; *p* < 0.01), and academic writing total (Z = −6.188; *p* < 0.01) It is seen that there is a statistically significant difference between the pre-and post-test scores. Considering the mean ranks (in favor of positive ranks), it can be said that this difference is in favor of post-test scores.

### Student’s reflective diaries for argumentation-based academic writing practices

After the academic writing practices in the first week and the eighth week, the students were not asked to keep a reflective diary. In the articles written in the first week, it was aimed for the students to see the shortcomings they experienced step by step and to transfer their progress to their diaries. As a result of their academic text writing practices in the eighth week, data were collected from the students through a semi-structured interview form. The students were asked to keep a diary in the activities implemented except for these 2 weeks.

In the second week of the implementation, it was aimed to define the concepts of academic writing and argumentation. At this stage, firstly, the elements of academic writing were conveyed and then the concept of argumentation was taught to the students. The similarities in the nature of academic research have been brought to the fore with the argumentation being based on concepts such as data-claim-justification. In this context, it has been discussed in the classroom environment that academic research is actually intertwined with the concept of argumentation, and how argumentation will contribute to the academic writing process. When the student diaries of the second week were examined, it was seen that the students stated that their knowledge about academic writing and argumentation was insufficient. Some of the students also put forward some thoughts about the inadequacy of the articles they wrote in the first week. Moreover, some of the students emphasized that they were far from these concepts and this situation created anxiety for them. The thoughts of the students about the second week practice were reflected in their diaries as follows:

*“Before class I realized that I had almost no knowledge of scientific writing. The things I knew weren’t very worthwhile, either. I saw my own shortcomings in the course.” (S17)*

*“I think I had some anxiety because I had never written an article before and because of the fear of not being able to do what I did not know.” (S35)*

*“I had never written an academic text before. So I had no idea about the subject. This is the first time I’ve heard of the argument. It cannot be said that I wrote the concepts of data-claim-justification very carefully.” (S25)*

*“I had not written a scientific article before and I did not know how to write a scientific article. This situation challenged me in my learning and writing process due to my inexperience.” (S53)*

*“I want to learn how to write articles better. The point I need to learn is how to create a more persuasive writing style, how to write a complete error-free article, how to provide more support to rebuttal views. I think this course contributed to that.” (S10)*

In the third week of the implementation, it is aimed to teach the use of literature review and some platforms that can be beneficial in this regard so that students can search for evidence for their arguments. First of all, information about the purposes of the literature review, the use of keywords and how to reach the right data were given. In this context, the use of Elsevier, ERIC, ULAKBİM and Google Scholar platforms, which can be beneficial to students in the context of educational sciences and Turkish education, were demonstrated in the classroom. When the student diaries were examined, it was seen that the students had no knowledge of Elsevier, ERIC and ULAKBİM platforms, and some of them had used the Google Scholar platform before. While some students stated that they used libraries as a source, some students emphasized that they benefited from various internet sites. The thoughts of the students about the third week practice were reflected in their diaries as follows:

*“Since I didn’t have much knowledge about this subject before the lesson, I realized that there were too many platforms to research.” (S47)*

*“I learned where and how I can reach reliable sources during the academic text preparation process. I learned platforms such as Google Scholar, ULAKBİM, Elsevier and ERIC, I think I can use these platforms more often now.” (S20)*

*“I did not know exactly how the literature review was done and what I should pay attention to. Thanks to this lesson, I learned how to do it. Since I did not know this before, I was worried about how to do it at first, but after getting informed, I saw that I could do it.” (S27)*

*“The reason why I had difficulty in searching the sources was that I had never done such serious research. In order to strengthen my claim in what I wrote, I did not look at different studies. While I used to only find and write on the website, now I am trying to reach information by reading articles.” (S42)*

*“I used to find resources by searching the literature on Google Scholar or going to the library. These were guiding my previous studies on the subject. I learned new ways in this lesson.” (S13)*

In the fourth week of the implementation, it was aimed to teach the citation rules and bibliography to the students. Citation is an element that contributes to the researcher in finding data and evidence to support his claims. Therefore, quoting correctly will contribute to both supporting the argument and improving academic writing skills. Since there are various standards in this regard, the teaching of the APA − 6 style, which is frequently used in the field of educational sciences, was preferred. When the students’ views on the practice were examined, it was seen that some students did not have much experience in citation and preparing bibliography, did not attach much importance to citation, and had difficulties in writing a bibliography. The views in the student diaries for the fourth week’s practice are as follows:

*“Before the lesson, I had no knowledge of the subject. I had no idea that when using someone’s word, we need to cite and write bibliography.” (S5)*

*“When citing, I learned where and how to specify it. I learned how to correctly specify any representation that will ensure the integrity of the text, such as the layout of the bibliography, etc.” (S29)*

*“During the course, I learned how important it is to prove a claim, opinion and defend it with a scientific study, by making references, and that the resources we have used on the subject will not be written randomly.” (S32)*

*“Through this course, I first learned how to write a correct article. I did not know much about citing, citation, and plagiarism before the lesson. Thanks to this lesson, I learned how to use these correctly in the article.” (S41)*

*“I knew about in-text citations before. So, it came easy to me. However, I had a hard time writing a bibliography because books, magazines, the internet, etc. in APA standards was different. I had some difficulty in learning because writing the bibliography of the articles was different. Of course, you don’t use them all in the same text.” (S21)*

In the fifth week of the implementation, studies were carried out to create a text for the argument presented by the student and to ensure consistency throughout the text. While creating an academic text, it is of great importance for the student to organize the text correctly and give intellectual patterns in logical order. In this way, both the argument will be defended consistently, and the academic writing will be made into an organized report. In this regard, some students’ difficulties were reflected in the statements in their diaries. Examples from the student diaries for the fifth week’s practice are as follows:

*“I had a hard time with this because I’m a beginner, I don’t have a good command of the objective language, and I tend to take topic in a different direction.” (S13)*

*“My arguments in the text I wrote today were qualified and convincing. the point I lacked was building a common bridge between the examples and having difficulty maintaining the objective language.” (S6)*

*“My goals were to express and prove my point of view in the best possible way, (words of important people, etc.) and I believe I could prove, and express them beautifully. I think I have organized the sentences and paragraphs correctly.” (S1)*

*“This course taught me how to defend and develop an idea when I come up with it because it is not enough to put forward the idea, it is necessary to convey it to the other party in a logical way.” (S9)*

*“Thanks to the arguments, we were providing our evidence to the alleged issues and strengthening their credibility. But we were writing irrelevant ideas while doing this. When you read too much, I think one can get distracted from the subject.” (S27)*

In the sixth week of the implementation, the topic was presenting the argumentative expression in academic texts with a scientific and objective attitude; thus studies have been carried out to use a fluent and understandable style in accordance with the rules of the language. The activity, which was carried out using sample texts in the classroom, aimed at equipping students with the ability to use the elements such as argumentative expression, scientific value, and objectivity, which are in the nature of argumentation and academic writing. When the student diaries regarding this week’s activity were examined, it was seen that the students understood the importance of argumentative expression, objective and scientific approach in an academic text and they want to improve themselves by doing exercises. Some of the statements taken from the student diaries written for the sixth week are as follows:

*“When I saw the difference between what I first wrote about argumentation and the work I do with my friends now, I saw that I was getting closer to my goals. I realized that I would be more beneficial to my students in the future by using punctuation marks and spelling rules better.” (S2)*

*“During the lesson, we learned how to write an article well, and how to use the language and expressions. Seeing different articles also contributed to this. Maybe we can learn to use a more objective language by taking other articles as an example.” (S16)*

*“We learned that the academic article is more formal and the article we write needs to be proven. I learned that we should not approach the issues that we tell ourselves or that our friends tell us with prejudice because it should not be forgotten that every subject can be true in terms of provability.” (S24)*

*“During the lesson, I learned the rules of a scientific writing, the steps of writing a scientific article, and how to defend our opinion while writing, and how to use argumentative speech to refute the opinion defended by the other party.” (S53)*

*“In order to write a better article, I needed to learn how to do a better literature review and, as a result, better defend my opinion with scientific data and write it in a more scientific language. I think today's lesson contributed to that.” (S18)*

In the seventh week of the implementation, the ideas of different authors in the literature and providing evidence for the argument put forward by the student were combined in the academic text in line with the student’s argument and a proofreading study was carried out. When the student diaries written about this activity were examined, it was seen that the students had difficulty in adding the statements that would support their arguments to their academic writing and experienced various concerns. In addition, it can be said that the proofreading study contributed to the writings of the students. Some statements taken from the student diaries written for the seventh week of the implementation are as follows:

*“I find it difficult to make sentences with my own words without plagiarizing the subject because it is difficult to put together sentences without being influenced. I'm worried about plagiarism.” (S22)*

*“I had difficulty in interpreting a person's thought in my own way and writing it down. I was worried and nervous. I was afraid of making mistakes.” (S48)*

*“When I read the text I wrote from beginning to end, and I saw that there were places that needed correction. I've been doing this in other articles I've written before. I have benefited from this habit.” (S45)*

*“I had never worked in an academic field before, or I used to call what we wrote as an article, but it turns out that it has nothing to do with it. Even before the writing phase, the teacher's explanation of the points that needed attention helped me to understand many of my shortcomings from the very beginning. With the activity we did in the last lesson, I can now write a better text.” (S7)*

*“Sometimes I had a hard time combining the quotations in the text because it was a little difficult for me to rephrase. But I think I have achieved that, especially by rereading.” (S19)*

### Student opinions on argumentation-based academic writing practice

**Figure 1:**
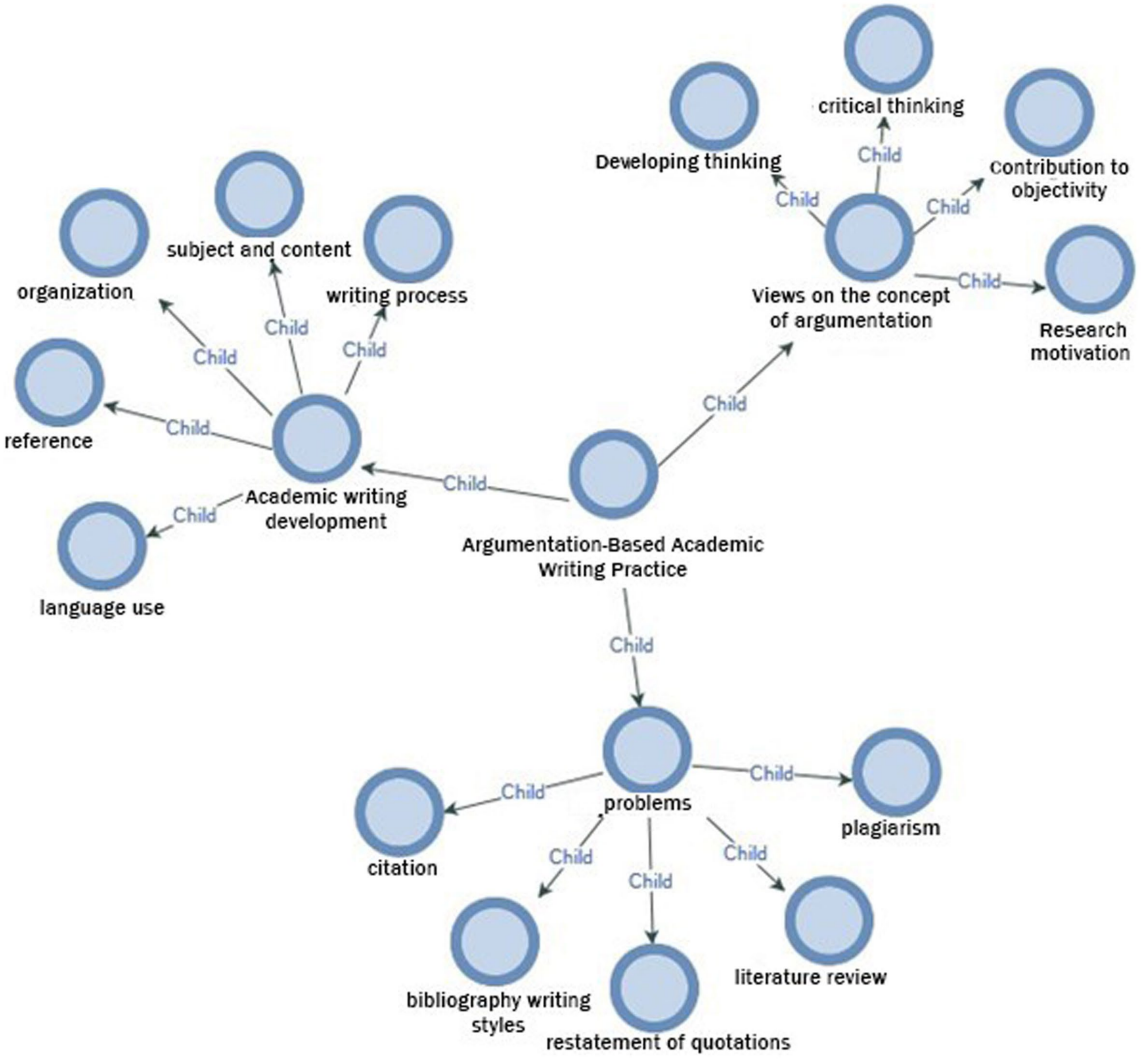
A model for student views on argumentation-based academic writing practice.

In this section, the findings obtained through the semi-structured interviews are included. The opinions of the students regarding the last research question, “What are the student views on argumentation-based academic writing practices?,” were gathered around three sub-themes: “the development of academic writing, the problems experienced, and the opinions on the concept of argumentation” (see Figure 1).

When the opinions related to the first sub-theme were examined, the statements of the students about the progress they made during the process of developing their argumentation-based academic writing skills were identified. The students’ expressions about their development were discussed under the headings of the subject and content, organization, citation, language use and writing process in the rubric used. The students discussed their development in the context of the process and expressed their views on the effects of the practices in question. Some student opinions on the contribution of the practices to the development of academic writing are as follows:

*“When we wrote a text around the subject we determined in our studies, I realized that I entered very different subjects in the first text. The main thing was to support the argument we determined without deviating from the topic. We used this along with the lessons we learned in our last article.” (S24)*

*“Considering the stages from the beginning to the end of the practice, I think that my academic writing proficiency has improved. I have made progress on the language and style to be used in writing an article. This is evident from the difference between the text when we first compose and the text at the end.” (S12)*

*“We actually use concepts such as logical order and organization in different articles. But I think I should have paid a little more attention as I was trying to prove my argument because in order to convince the other side, it is necessary to construct a logical article.” (S44)*

*“I think there has been improvement in my academic writing proficiency because I had a hard time citing and writing bibliography at first, but now I can do it without much difficulty.” (S37)*

*“I have never done proofreading in the texts I have written before. There may be places that need editing in the text, especially in an academic text, there should be no errors. In this respect, I see it as a positive aspect of the implementation.” (S34)*

When the opinions of the second sub-theme are examined, it was seen that the students drew attention to some difficulties they experienced during the activities for the development of argumentation-based academic writing processes. Elements such as citation and bibliography writing styles, restatement of quotations, and plagiarism, which were seen for the first time for some of the students, brought along certain difficulties. Some of the students’ views on the elements they had difficulties in the implementation process are as follows:

*“I especially had problems in citing and plagiarism. because I had never defended a topic with such detail and based on evidence in any study before. What’s more, I thought what if it was wrong, because their correct use is also important.” (S7)*

*“Like every new learner, I experienced difficulties. I had trouble citing because this step was a little more detailed. It was wrong to write the bibliography as I wrote the websites as they are, but I tried to edit them later and write them correctly.” (S19)*

*“I had problems with plagiarism at the beginning and the end of the implementation. You have trouble finding the appropriate words when conveying someone else's opinion with your own. I try to use synonyms, but I don't know if that's right or wrong." (S24)*

*“I just had a hard time doing a literature review. I realized that I didn't know how to search for the topic. When I wrote the subject, I thought it would be right in front of me. I learnt that it was not like that, there were different platforms." (S49)*

*“I had some difficulties in the citation and bibliography writing at the beginning because I was hesitant about whether I got it directly or from the citation in the in-text citations. In the bibliography writing, there were various writing styles such as magazines, internet, and periodicals. So, I had some trouble.” (S12)*

When the opinions of the third sub-theme are examined, the statements of the students about the concept of argumentation stand out. By emphasizing that argumentation contributes to academic writing, students highlighted its contribution to thinking and developing thinking skills. In addition, the students stated that the desire to defend an idea and refute the counter-idea provided more research motivation. Furthermore, it was revealed by some students that the effort to refute the counter-idea improved their critical thinking skills and that defending the idea by making use of different studies added objectivity to the academic article. Some student views on the concept of argumentation are as follows:

*“Argumentation makes even obsessed people think on occasion. It enables people to look from different perspectives, develop their thoughts with different thoughts, and get rid of the stereotypes.” (S1)*

*“I think that since the argumentation-based teaching practice reminds us of the importance of research and teaches that not every source is the right source, the information you have obtained from the internet and here can actually be empty, so I think that the practice should be done by every educator.” (S31)*

*“I find it important to do research in the argumentation-based teaching process, to know different views, to research the subject from different sources, to reach real information and to verify it. I loved doing research from different sources and sites.” (S5)*

*“I find the activities in the argumentation-based teaching process useful because we need to base our claims on justifications in order to believe the subject we are defending, and we need to research it further. In this way, permanent learning is provided, and the subject is comprehended more. This gives us motivation to investigate further.” (S14)*

*“While trying to prove and document that the claims of the other party are false during the writing process, we think critically and approach the opinions of the other party in a questioning manner, and we can freely express their opinions.” (S29)*

## Conclusion and discussion

Considering the results of the research, it was determined that the arithmetic mean of the scores that the students got from the academic texts they wrote before the argumentation-based academic writing practices were carried out was 2,256. The score increased to 3,554 in the academic texts written after the implementation. When the pretest-posttest scores of the students are examined in the context of the dimensions in the rubric, it is seen that there is a significant difference in all of the dimensions, namely “subject and content,” “organization,” “language use,” “citation” and “writing process.” It can be said that the implementation carried out in this research had a positive effect on the academic text writing skills of the students. Indeed, the reflections of this development can be seen in the qualitative data collected during the research process. At the end of the activities, some students’ opinions came to the forefront that they could write the text they wrote within the framework of the arguments they put forward, organize the ideas in the text in a logical order, use a more scientific and objective language, apply the rules of citation and bibliography, integrate different ideas in their writing, and do proofreading. These views support the quantitative data. When the academic texts written before the implementation are examined, it is seen that the students had various deficiencies in writing academic texts. As a matter of fact, in other studies conducted with Turkish teacher candidates, the findings have also revealed that some students did not understand the language used in scientific texts (Yücelşen and ve Edizer, 2020), made mistakes in writing planning, had difficulty in finding the main idea and supporting idea (Arıcı, 2008), and could not provide logical integrity in the text (Göçer, 2010). Moreover, most of the Turkish teacher candidates who participated in the research conducted by Yücelşen and ve Edizer (2020) emphasized that they had difficulties in this regard because they had not practiced academic writing before. The aforementioned research results coincide with the pre-implementation statements of our study group. In our research, student diaries were used to obtain data on the implementation process. When the diaries were examined, it was seen that the students did not have enough knowledge about the concepts of academic writing and argumentation, but they gradually gained knowledge as the weeks progressed. In this process, the concepts of academic writing and argumentation were introduced, the information on literature review was given and the use of various platforms, citation and bibliography rules were taught. A diary was kept by the students during the implementation phase of the study, and weekly developments were observed based on the expressions in the diaries. These developments were also supported by quantitative data and student statements obtained from the semi-structured interviews.

Although there are significant differences in the development of students in the argumentation-based academic writing process, it was stated by the students that there are problems at some points. While some of these problems were reflected in the diaries during the implementation process, some of them continued as a result of the implementation and were expressed in the interviews. The topics that the students expressed their difficulties experienced during the process only in the diaries they kept were identifies as the consistency of subject and content, the organization of the text, the use of language and proofreading. When the studies in the literature are examined, it is seen that some students cannot use the information in the literature for a purpose (Groom, 2000, p. 67), they cannot relate the evidence they reach with their claims (Watson et al., 2004), they write statements irrelevant to their topic; they give irrelevant information, and they cannot express the ideas in a logical order (Wingate, 2012). They feel inadequate in their written expression skills (Bağcı, 2007), and they do not practice proofreading (Yücelşen and ve Edizer, 2020). In this context, it is acceptable for some students to experience difficulties in these topics during the education process. In addition, based on the interviews conducted at the end of the implementation, the continuing difficulties in the academic writing processes of the students were identified as the methods of citation and bibliography writing styles, restatement of citations, plagiarism, and use of recommended platforms for literature review. At the end of the implementation, learning the citation, and bibliography writing styles were expressed by the students as the most difficult subjects to master. The aforementioned student statements can also be found in the quantitative research results, and it is seen that the least developed topic is “citation.” Similarly, in another study, it was revealed that more than half of the Turkish language teaching students participating in the research conducted by Yücelşen and ve Edizer (2020) do not know that they should indicate the sources they use, and that there is no student who knows the correct way of citing sources. In addition, the fact that Google Scholar was preferred among the platforms used by the students in the literature review process and that ULAKBİM, Elsevier or ERIC platforms were not preferred can be seen as supporting data for similar study groups. Another essential element for writing an academic text is to exhibit good language and expression skills. Authors can synthesize and present texts from different research in their studies, and can write shorter versions by restating long texts. Thus, space for more data will be provided in the study and the problems that may rise regarding plagiarism will be minimized (Kozak, 2018). However, it is noteworthy that some students in our study group stated that they had difficulties in these matters though it was the end of the implementation period.

An academic paper in the social sciences requires the author to make a claim about a subject and support this claim with evidence to persuade the reader (Wood, 2001). In this context, it can be said that the concepts of argumentation and academic writing are intertwined. As a result of the argumentation-based academic writing practices based on this idea, it was revealed that the students put forward various opinions about the concept of argumentation. The students participating in the research emphasized that argumentation contributes to academic writing and highlighted its contribution to thinking and developing thinking skills. In this context, Doğan (2012) emphasized that in educational discussion activities, basing claims on evidence is important in providing students with a different perspective. In different studies in the literature, it has been stated that argumentation contributes to the development of high-level thinking and reflective thinking, judgment and reasoning skills (Nussbaum, 2002; Erduran and Jimenez-Aleixandre, 2007; Kosko et al., 2014; Antonio, 2020). Besides, the students claimed that the desire to defend an idea and refute the counter-idea in academic writings provides motivation for doing more research. These thoughts coincide with the data in the literature that argumentation arouses a sense of curiosity, develops research-inquiry skills, directs the individual to research and motivates him/her (Apaydın and Kandemir, 2018; Özcan et al., 2018; Karaer et al., 2019). Within the scope of the present research, some students stated that argumentation contributed to the development of critical thinking skills. The approach of supporting a claim with evidence and refuting the counter-idea, which is inherent in argumentation, requires critical thinking. Within the framework of this necessity, the contribution of argumentation to critical thinking skills has been revealed in different studies (Namdar and Salih, 2017; Giri and Paily, 2020). In addition, it was stated by some students that defending the argument by making use of different studies added objectivity to an academic article. Argumentation encourages students to use scientific theories, data and evidence to defend their claims about a topic (Simon et al., 2006). The use of different scientific data in an academic article will contribute to more objective expressions in that article.

When the results of the research are considered in general, it was seen that academic writing practices based on argumentation contributed to the development of students in the sub-dimensions of “subject and content,” “organization,” “language use,” “citation” and “writing process.” In the data obtained from reflective diaries and interviews, although some difficulties were pointed out, the statements of the students regarding their development came to the fore. In addition, it was emphasized that argumentation contributed not only to academic writing skills, but also to development of thinking, objectivity, research motivation and critical thinking.

Based on the results of the study, the following suggestions can be made:

1. Considering the mistakes of the students in the texts they first wrote, it can be said that they did not have enough information about the elements of academic writing, so they did not have enough information in the previous stages. In this context, argumentation-based activities can be carried out to improve students’ academic writing skills, especially in activities related to informative texts at pre-graduate education levels.
2. The research was carried out with undergraduate level participants who received Turkish language teaching education. The effect of argumentation in academic writing training in different teaching areas can be examined.
3. Considering the student scores before the implementation, it was seen that the academic writing skills of the students should be improved. In order to fulfil this requirement, requirement, besides argumentation-based education in academic writing education, different education programs can be prepared, and the effectiveness of these programs can be examined comparatively.
4. In the statements that emerged in the semi-structured interviews with the students, it was stated that argumentation is an improvement in the areas of development of thinking skills, objectivity, research motivation and critical thinking. In future studies, the relationship between these concepts can be examined.

## Data availability statement

The raw data supporting the conclusions of this article will be made available by the authors, without undue reservation.

## Ethics statement

The studies involving human participants were reviewed and approved by Akdeniz University Rectorate Social and Human Sciences Scientific Research and Publication Ethics Committee. The patients/participants provided their written informed consent to participate in this study.

## Author contributions

BD, SA, and BŞ design of the study and wrote sections of the manuscript. MG and ENÇ organized the database and performed the quantitative and qualitative analysis. BD, SA, BŞ, MG, and ENÇ contributed to the manuscript revision. All authors read and approved the submitted version.

## Conflict of interest

The authors declare that the research was conducted in the absence of any commercial or financial relationships that could be construed as a potential conflict of interest.

## Publisher’s note

All claims expressed in this article are solely those of the authors and do not necessarily represent those of their affiliated organizations, or those of the publisher, the editors and the reviewers. Any product that may be evaluated in this article, or claim that may be made by its manufacturer, is not guaranteed or endorsed by the publisher.
